# Aberrant Alternative Splicing in U2af1/Tet2 Double Mutant Mice Contributes to Major Hematological Phenotypes

**DOI:** 10.3390/ijms22136963

**Published:** 2021-06-28

**Authors:** Cristina Martínez-Valiente, Cristian Garcia-Ruiz, Beatriz Rosón, Alessandro Liquori, Elisa González-Romero, Raúl Fernández-González, Isabel Gómez-Redondo, José Cervera, Alfonso Gutiérrez-Adán, Alejandra Sanjuan-Pla

**Affiliations:** 1 Hematology Research Group, Instituto de Investigación Sanitaria La Fe, Avda. Fernando Abril Martorell 106, 46026 Valencia, Spain; cristina_martinez@iislafe.es (C.M.-V.); cristian_garcia_ruiz@iislafe.es (C.G.-R.); beatriz.roson@gmail.com (B.R.); alessandro_liquori@externos.iislafe.es (A.L.); elisa_gonzalez@iislafe.es (E.G.-R.); 2 Animal Reproduction Department, INIA, Ctra. de La Coruña, km 7.5, 28040 Madrid, Spain; raulfg@inia.es (R.F.-G.); igomer00@gmail.com (I.G.-R.); agutierr@inia.es (A.G.-A.); 3 Hematology Service, Hospital Universitario y Politécnico La Fe, Avda. Fernando Abril Martorell 106, 46026 Valencia, Spain; cervera_jos@gva.es; 4 Centro de Investigación Biomédica en Red de Cáncer (CIBER-ONC), Av. Monforte de Lemos, 3-5 Pabellón 11, 28029 Madrid, Spain; 5 Genetics Unit, Hospital Universitario y Politécnico La Fe, Avda. Fernando Abril Martorell 106, 46026 Valencia, Spain

**Keywords:** myelodysplastic syndrome, U2AF1, TET2, alternative splicing

## Abstract

Mutations in splicing factors are recurrent somatic alterations identified in myelodysplastic syndromes (MDS) and they frequently coincide with mutations in epigenetic factors. About 25% of patients present concurrent mutations in such pathways, suggesting a cooperative role in the pathogenesis of MDS. We focused on the splicing factor U2AF1 involved in the recognition of the 3′ splice site during pre-mRNA splicing. Using a CRISPR/Cas9 system, we created heterozygous mice with a carboxy-terminal truncated U2af1 allele (U2af1^mut/+^), studied the *U2af1*^mut/+^ hematopoietic system, and did not observe any gross differences in both young (12–13 weeks) and old (23 months) *U2af1*^mut/+^ mice, except for a reduction in size of approximately 20%. However, hematopoietic stem/progenitor cells lacked reconstitution capacity in transplantation assays and displayed an aberrant RNA splicing by RNA sequencing. We also evaluated *U2af1*^mut/+^ in conjunction with Tet2-deficiency. Novel double mutant *U2af1*^mut/+^ *Tet2*^−/−^ mice showed increased monogranulocytic precursors. Hematopoietic stem/progenitor cells were also enhanced and presented functional and transcriptomic alterations. Nonetheless, *U2af1*^mut/+^ *Tet2*^−/−^ mice did not succumb to MDS disease over a 6-month observation period. Collectively, our data suggest that cooperation between mutant U2af1 and Tet2 loss is not sufficient for MDS initiation in mice.

## 1. Introduction

Myelodysplastic syndromes (MDS) comprise clonal hematological diseases that appear more commonly in the elderly and that can adversely evolve into secondary acute myeloid leukemia (sAML) in up to 40% of patients. In the last decade, next-generation sequencing technologies (NGS) have largely deciphered the molecular genetics of MDS. Most frequently mutated genes (>50%) codify for splicing factors, mainly *SF3B1, SRSF2, U2AF1,* and *ZRSR2* [1,2]. Alternative splicing is mostly executed by the major spliceosome, a complex RNA and protein machinery that catalyzes the splicing reaction. The U2 Small Nuclear RNA Auxiliary Factor 1 (U2AF1) forms a heterodimer with U2AF2 to constitute U2AF, and U2AF1 recognizes the 3′ AG dinucleotide at the 3′ splice site (SS) of a pre-mRNA intron, resulting in the subsequent recruitment of U2 small nuclear ribonucleoprotein (U2-snRNP) during pre-mRNA splicing. Mutations in *U2AF1* are present in patients with MDS (in approximately 11% of cases), myeloproliferative neoplasms (MPN), clonal hematopoiesis of indeterminate potential (CHIP), acute myeloid leukemia (AML, in about 4% of patients), chronic lymphocytic leukemia (CLL), and a variety of solid tumors [1]. The most prevalent *U2AF1* mutations are located at two hotspots, and they affect the S34 and Q157 residues within the zinc finger domains (Zn) [3]. Occasionally, rare non-hotspot *U2AF1* mutations are found as missense or indel changes [4]. *U2AF1* mutations are heterozygous and are associated with adverse prognosis, increased risk of progression to AML, and poor overall survival (OS) in MDS and MPN patients [5], with U2AF1^Q157^ having a worse OS than U2AF1^S34^ [6]. Currently, it is unclear whether *U2AF1* mutations result in a gain-of [7,8] or loss-of-function of the protein [5,9]. Importantly, mutated *U2AF1* requires the presence of a wild-type allele for surviving and counteracting aberrant splicing [8,10].

*U2AF1* mutations can contribute to MDS disease pathogenesis through different mechanisms, such as mRNA translation [11], R loop formation [12,13], and mis-splicing induction [14]. RNA sequencing has revealed that *U2AF1* mutations affect exon skipping [3], but when U2AF1^S34F^ and U2AF1^Q157P^ are compared, no substantial overlap in alternatively spliced targets is observed [15]. Additionally, functional studies in *U2AF1*^S34F^ and *U2AF1*^Q157P^ doxycycline-inducible transgenic mice demonstrated distinct effects on RNA splicing and hematopoiesis, suggesting that these mutants may affect MDS differently [16,17]. While the functional impact of the more frequent U2AF1^S34F^ mutation has been largely studied [9,13,18,19,20], the effects of U2AF1^Q157^ are less explored. Additionally, mice with conditional loss of *U2af1* in the hematopoietic system die from severe aplasia, demonstrating that *U2af1* is essential for hematopoietic stem cells (HSC) survival and function. In addition, no phenotype was detected in *U2af1* heterozygous knock-out mice (*U2af1*^+/−^) [8,21]. Of note, the concurrence of *U2AF1* mutations with those in epigenetic regulators, such as *ASXL1* and *TET2*, has been described in MDS patients [5,22,23]. In mice, AML development has been observed in knock-in animals carrying a *U2af1^S34F^* mutation in combination with *Runx1* deficiency and predicted loss-of-function *Tet2* mutations [20].

TET2 catalyzes the oxidation of 5-methylcytosine (5mC) to 5-hydroxymethyl-cytosine (5hmC) and other oxidation products, promoting DNA demethylation. *TET2* is the most frequently mutated gene in MDS [24], and biallelic *TET2* gene inactivation is frequently observed in myeloid neoplasms [25]. Mutations in *TET2*, with a usually low variant allele frequency (VAF), are also found in CHIP [26]. At the cellular level, TET2 is involved in the regulation of HSC self-renewal and myeloid lineage commitment [27]. *Tet2* hematopoietic conditional knock-out mice result in a prolonged expansion of HSC, with skewed differentiation towards monocytic progenitors, splenomegaly, and extramedullary hematopoiesis with an overall mild phenotype [28,29]. Although combined mutations in *TET2* and splicing factors are thought to give rise to MDS and MPN, the pathogenic mechanisms underlying concurrent mutations have not been described previously. In the present study, we investigated the function of mutant *U2af1* on hematopoiesis in a novel CRISPR/Cas9 mouse model and assessed the impact of the concurrent disruption of *U2af1* and *Tet2* on the pathogenesis of MDS.

## 2. Results

### 2.1. Development of U2af1 Mutant Allele

**Figure 1:**
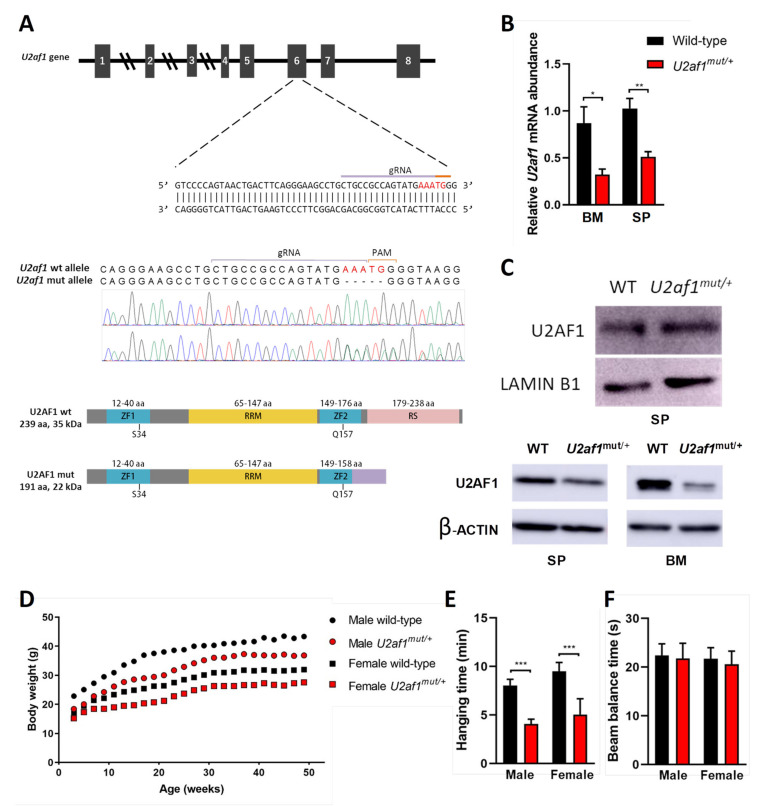
Generation of U2af1 mutant mouse. (**A**) Upper panel: gRNA sequence against exon 6 of murine U2af1 gene. Middle panel: Sanger sequencing chromatograms in WT and U2af1^mut/+^ genomic DNA. Five-nucleotide deletion is indicated in red. Lower panel: WT and truncated U2AF1 protein upon CRISPR-Cas9 gene edition. According to the primary sequence, the kDa value of the truncated protein was calculated using the ExPASy ProtParam tool. ZF: Zinc Finger; RRM: RNA recognition motif; RS: arginine-serine rich domains. (**B**) Relative mRNA abundance of U2af1 gene for WT and U2af1^mut/+^ mice quantified by RT-qPCR in bone marrow (BM) and spleen (SP). Data normalized to β--actin. (BM: WT *n* = 5, U2af1^mut/+^ *n* = 4; SP: WT *n* = 6, U2af1^mut/+^ *n* = 5). (**C**) Upper part: Western blot of U2AF1 using a N-terminal antibody on spleen cell lysates. Lamin B1 was used as housekeeping control (WT *n* = 4, U2af1^mut/+^ *n* = 4). Bottom part: Western blot of U2AF1 using a C-terminal antibody on spleen and bone marrow cell lysates. β-Actin was used as housekeeping control (WT *n* = 2, U2af1^mut/+^ *n* = 2). (**D**) Body weight of male (*n* = 14) and female (*n* = 18) U2af1^mut/+^ versus male (*n* = 12) or female (*n* = 16) WT mice during adulthood. (**E**) Neuromuscular strength assessed by wire hang test in male or female mutant (red bar) and WT mice (black bar) at 9 months (*n* = 12–14/group). (**F**) Beam balance test in WT and U2af1^mut/+^ mice (*n* = 10/group) at 4 and 10 months of age. (**B**–**E**) Mean values ± SEM are represented. Asterisks indicate significant changes by unpaired Student’s t test (* *p* < 0.05; ** *p* < 0.01; *** *p* < 0.001).

To determine the effect of U2af1 on hematopoietic development, we genetically manipulated the second Zn that affects all the described *U2af1* isoforms [30,31] to create a mutant truncated allele at the C-terminus by a CRISPR−Cas9 system (Figure 1A). We first analyzed the 5-nucleotide deletion in the DNA of control WT and heterozygous *U2af1^mut/+^* mice by Sanger sequencing (Figure 1A). At the transcript level, wild-type *U2af1* mRNA expression in bone marrow and spleen of heterozygous *U2af1^mut/+^* was decreased in comparison to control WT mice (Figure 1B). At the protein level, no band corresponding to the truncated allele was observed in heterozygous *U2af1^mut/+^* samples. This could be because the mutant transcript is very lowly expressed; alternatively, the translated mature protein could be degraded by the proteasome or might not be detected with our N-terminal antibody due to conformational or stability issues (Figure 1C). When an antibody targeting the RS region was used, a decrease in U2af1 levels was observed in heterozygous *U2af1*^mut/+^ samples. Homozygous *U2af1* mice, with both alleles edited, reached the blastocyst stage but died afterwards. Heterozygous *U2af1*^mut/+^ mice were viable but presented smaller size and developmental abnormalities with delayed reproductive maturity. As shown in Figure 1D,E, size of animals, growth, and muscle strength were affected in heterozygous *U2af1*^mut/+^ mice in comparison to WT animals. No differences were found in the equilibrium, as assessed by the beam balance test (Figure 1F).

### 2.2. Old U2af1 Mutant Mice Show Normal Hematopoiesis and Uneven Splenomegaly

Given that MDS clinically manifests at an advanced age, we analyzed 23 months old heterozygous *U2af1*^mut/+^ and control WT mice. Blood counts revealed no peripheral cytopenias (Figure S1a,b) and morphological analysis showed no signs of dysplasia (Figure S1c). Flow cytometry analysis of stem and progenitor bone marrow populations revealed no significant differences between *U2af1*^mut/+^ and WT mice (Figure S1d). Notably, uneven splenomegaly was observed in some *U2af1*^mut/+^ mice (Figure S1e). Collectively, we did not observe cardinal features of myelodysplasia in *U2af1*^mut/+^ heterozygous old mice.

### 2.3. Monogranulocytic Progenitors Are Increased in U2af1^mut/+^ Tet2^−/−^ Mutant Mice

**Figure 2:**
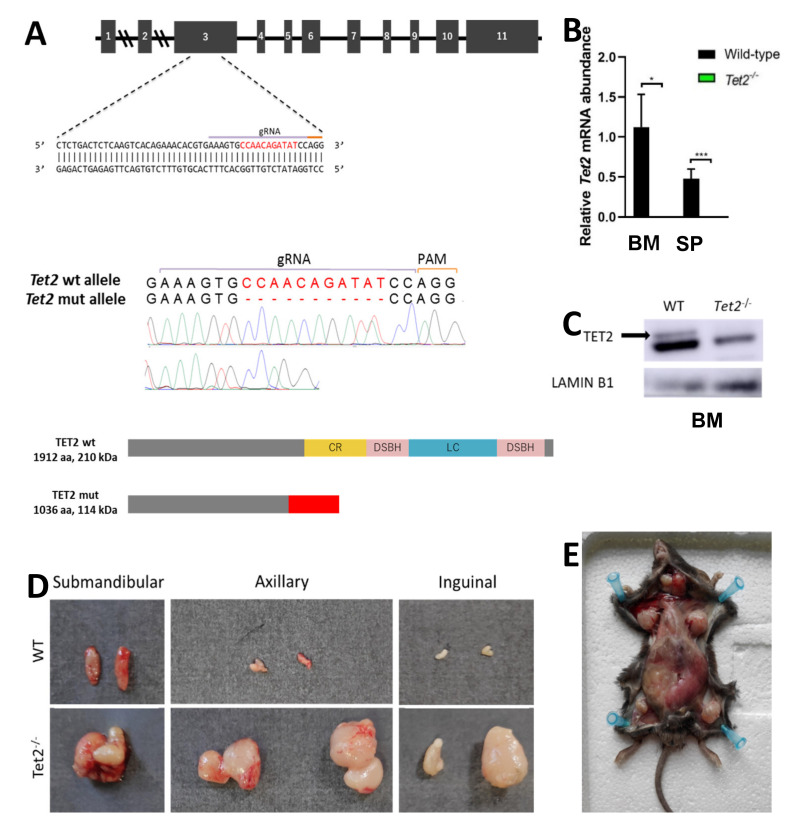
Generation of Tet2 mutant mice. (**A**) Upper panel: gRNA sequence against exon 3 of murine Tet2 gene. Middle panel: Sanger sequencing chromatograms of WT and Tet2^−/−^ mouse genomic DNA. Eleven-nucleotide deletion is indicated in red. Lower panel: WT and truncated TET2 protein upon CRISPR-Cas9 gene edition. According to the primary sequence, kDa value of the truncated protein was calculated using the ExPASy ProtParam tool. (**B**) Relative mRNA abundance of Tet2 gene for WT and Tet2^−/−^ mice quantified by RT-qPCR in bone marrow (BM) and spleen (SP). Data normalized to Hprt (BM: WT *n* = 6, Tet2^−/−^ *n* = 7; SP: WT *n* = 4, Tet2^−/−^ *n* = 7; * *p* < 0.05; *** *p* < 0.001). (**C**) Western blot of TET2 on bone marrow cell lysates. Lamin B1 was used as housekeeping control (WT *n* = 2, Tet2^−/−^ *n* = 2). (**D**) Gross morphology of submandibular, axillar and inguinal lymph nodes from representative WT (upper panel) and Tet2^−/−^ (lower panel) age-matched mice. (**E**) General view of Tet2^−/−^ mice’s anatomy, showing a significant enlargement of the spleen and the lymph nodes.

Mutations in epigenetic modifiers and splicing factors commonly co-occur in MDS, including *TET2* loss-of-function mutations and *U2AF1* mutations [23]. We generated a novel *Tet2*^−/−^ mouse model by CRISPR/Cas9 to study the concurrence with *U2af1*. We crossed heterozygous *U2af1*^mut/+^ mice with *Tet2* knock-out mice (Figure 2A). At the DNA level, an 11-nucleotide deletion was confirmed by Sanger sequencing in homozygous *Tet2*^−/−^ mice (Figure 2A). Lack of expression of wild-type *Tet2* in *Tet2*^−/−^ mice was observed at the mRNA level (Figure 2B) and at the protein level (Figure 2C).

**Figure 3:**
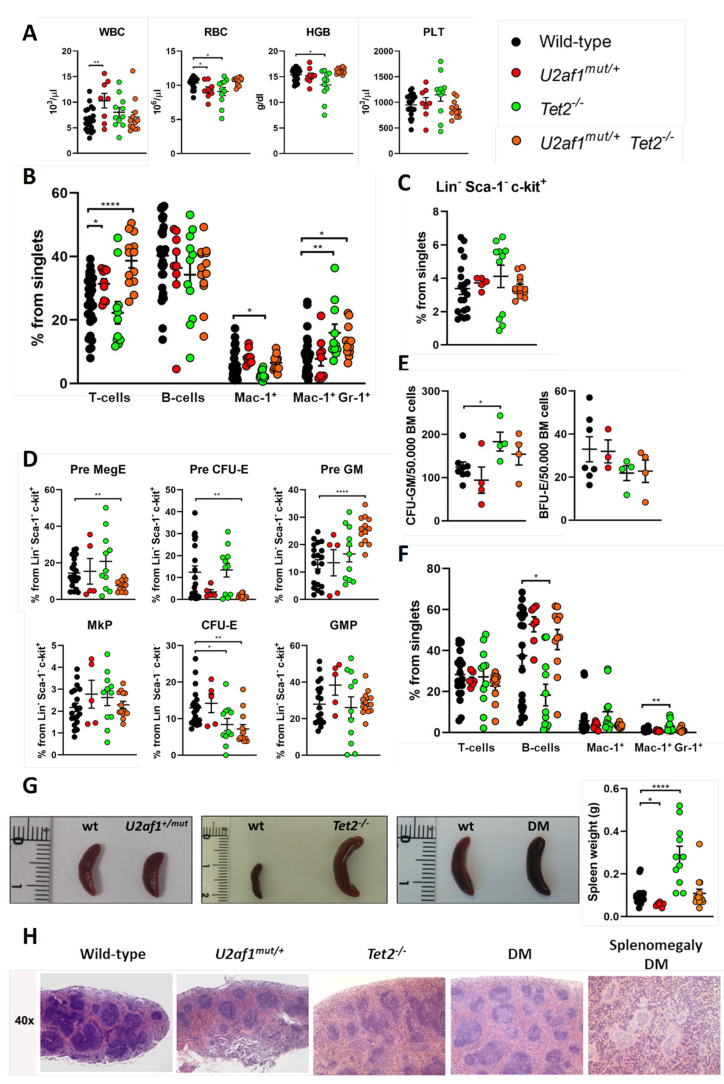
Hematological characterization of U2af1^mut/+^ Tet2^−/−^ mice. (**A**) Peripheral blood counts at 8–39 weeks (WT *n* = 22, U2af1^mut/+^ *n* = 8, Tet2^−/−^ *n* = 11, U2af1^mut/+^ Tet2^−/−^ *n* = 12). (**B**) Frequencies of peripheral blood mature cells at 8–39 weeks (WT *n* = 30, U2af1^mut/−^ *n* = 9, Tet2^−/−^ *n* = 11, U2af1^mut/+^ Tet2^−/−^ *n* = 13). (**C**) Frequencies of progenitors (Lin^-^ Sca-1^−^ c-kit^+^) in bone marrow (WT *n* = 20, U2af1^mut/+^ *n* = 5, Tet2^−/−^ *n* = 11, U2af1^mut/+^ Tet2^−/−^ *n* = 13). (**D**) Myelo-erythroid progenitors as percentage of progenitor cells at 8–39 weeks (WT *n* = 20, U2af1^mut/+^ *n* = 5, Tet2^−/−^ *n* = 11, U2af1^mut/+^ Tet2^−/−^ *n* = 13). (**E**) CFU-GM (WT *n* = 8, U2af1^mut/+^ *n* = 4, Tet2^−/−^ *n* = 4, U2af1^mut/+^ Tet2^−/−^ *n* = 4) and BFU-E (WT *n* = 7, U2af1^mut/+^ *n* = 3, Tet2^−/−^ *n* = 4, U2af1^mut/+^ Tet2^−/−^ *n* = 4) in unfractionated BM at 12–28 weeks. (**F**) Frequencies of splenic mature cells at 8–39 weeks (WT *n* = 21, U2af1^mut/+^ *n* = 7, Tet2^−/−^ *n* = 11, U2af1^mut/+^ Tet2^−/−^ *n* = 11). (**G**) Images of spleens and quantification of spleen weight at 8–39 weeks (WT *n* = 23, U2af1^mut/+^ *n* = 7, Tet2^−/−^ *n* = 11, U2af1^mut/+^ Tet2^−/−^ *n* = 14). (**H**) Representative histological images of spleen sections of mice at 12–14 weeks (WT *n* = 2, U2af1^mut/+^ *n* = 1, Tet2^−/−^ *n* = 1, U2af1^mut/+^ Tet2^−/−^ *n* = 2). (**A**–**G**) Mean values ± SEM are represented. Asterisks indicate significant changes by unpaired Student’s t test (* *p* < 0.05; ** *p* < 0.01; **** *p* < 0.0001).

We performed comprehensive analyses of the hematopoietic system in WT, single and double mutant mice. No differences were detected in blood parameters of double mutant mice in comparison to control (Figure 3A). In peripheral blood, *U2af1*^mut/+^ *Tet2*^−/−^ mice showed an increase in T cells (mean, 38.66% *U2af1*^mut/+^ *Tet2*^−/−^ versus 25.20% WT, *p* < 0.0001) and in monogranulocytic (Mac-1^+^Gr-1^+^) cells (mean, 13.06% *U2af1*^mut/+^ *Tet2*^−/−^ versus 8.71% WT, *p* < 0.05) (Figure 3B). Bone marrow analysis of Lin^-^Sca-1^-^c-kit^+^ progenitors showed no differences among genotypes (Figure 3C). Within myelo-erythroid progenitors, *U2af1*^mut/+^ *Tet2*^−/−^ mice showed a decreased percentage of precursors of bipotent megakaryocytic-erythroid progenitors (Pre MegE) (mean, 7.58% *U2af1*^mut/+^ *Tet2*^−/−^ versus 14.42% WT, *p* < 0.01) and precursors of erythroid progenitors (Pre CFU-E and CFU-E) (mean, 1.45% *U2af1*^mut/+^ *Tet2*^−/−^ versus 12.39% WT, *p* < 0.01) but an enhanced percentage of precursors of granulocyte-monocyte progenitors (Pre GM) (mean, 25.27% *U2af1*^mut/+^ *Tet2*^−/−^ versus 12.80% WT, *p* < 0.0001) (Figure 3D). This was accompanied by a non-significant increased CFU-GM and decreased BFU-E colonies in *U2af1*^mut/+^ *Tet2^−/−^* mice (Figure 3E).

In spleen, flow cytometry analysis confirmed an amplification of myeloid cells in *Tet2*^−/−^ mice, with a significant increase in Mac-1^+^Gr-1^+^ population (mean, 3.02% *Tet2*^−/−^ versus 1.06% WT, *p* < 0.01) (Figure 3F). This was accompanied by splenomegaly in *Tet2*^−/−^ mice that manifested with incomplete penetrance (Figure 3G). These animals also showed marked lymphadenopathies, with enlarged submandibular, axillar, and inguinal lymph nodes (Figure 2D,E). Microscopical examination of spleens in double mutant mice showed splenomegaly with disruption of white and red pulp structure in 1 out of 11 cases (Figure 3G,H). In all, data suggest that *U2af1*^mut/+^ *Tet2*^−/−^ progenitors are skewed towards myeloid lineage at expenses of the erythroid lineage.

### 2.4. U2af1^mut/+^ and U2af1^mut/+^ Tet2^−/−^ Mutant HSPC Show a Defect in Reconstitution Capacity

**Figure 4:**
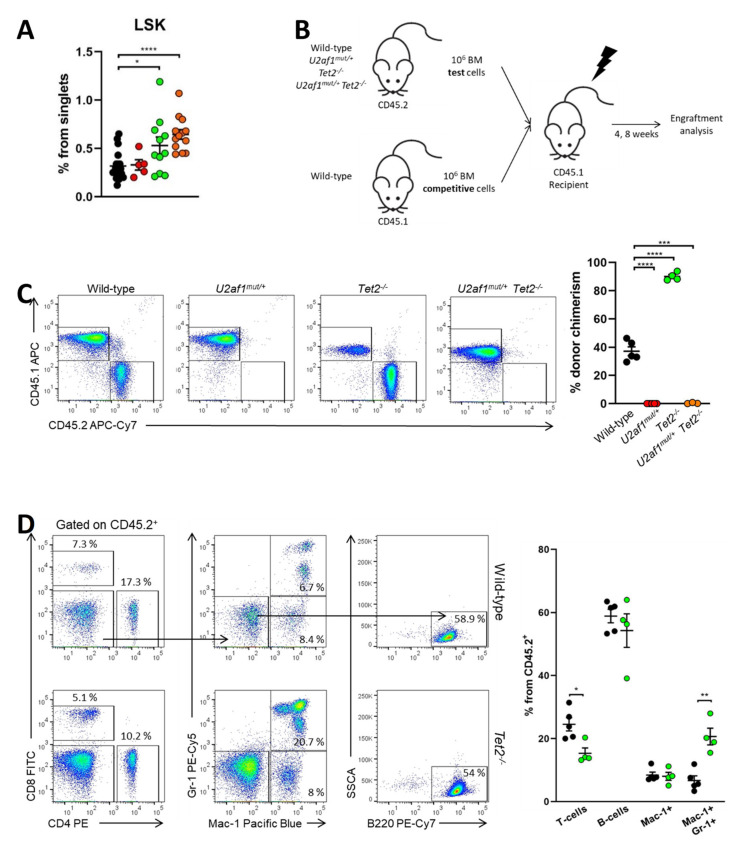
*U2af1^mut/+^*and *U2af1^mut/+^ Tet2^−/−^* HSPC show a defect in repopulation capacity. (**A**) Frequencies of LSK (Lin^-^ Sca-1^+^ c-kit^+^) in the bone marrow from WT and *U2af1^mut/+^* mice (WT *n* = 20, *U2af1^mut/+^ n* = 5, *Tet2*^−/−^ *n* = 11, *U2af1^mut/+^ Tet2*^−/−^ *n* = 13). (**B**) Scheme of competitive transplantation assay. (**C**) Peripheral blood CD45.2^+^ donor chimerism at 8 weeks post-transplantation (WT *n* = 5, *U2af1^mut/+^ n* = 5, *Tet2*^−/−^ *n* = 4, *U2af1^mut/+^ Tet2*^−/−^ *n* = 3). (**D**) Multilineage engraftment at 8 weeks post-transplantation in WT and *Tet2*^−/−^ transplanted mice. Plots show the percentage of myeloid (Mac-1^+^ Gr-1^+^), B (B220^+^) and T (CD4^+^ CD8^+^) within engrafted CD45.2^+^ cells (WT *n* = 5, *Tet2^−/−^ n* = 4). (**A**–**D**) Mean values ± SEM are represented. Asterisks indicate significant changes by unpaired Student’s t test (* *p* < 0.05; ** *p* < 0.01; *** *p* < 0.001; **** *p* < 0.0001).

We sought to determine whether these changes were due to the effects of the mutant splicing factor on blood stem cells. At the stem/progenitor compartment, LSK (marked by Lin^−^Sca-1^+^c-Kit^+^) cells were increased in *U2af1^mut/+^ Tet2^−/−^* mice (mean, 0.65% *U2af1*^mut/+^ *Tet2*^−/−^ versus 0.32% WT, *p* < 0.0001) (Figure 4A). To test hematopoietic function in vivo, we competitively transplanted 1:1 ratios of donor (WT, *U2af1^mut/+^, Tet2^−/−^* or *U2af1^mut/+^ Tet2^−/−^*; CD45.2) and competitor (WT; CD45.1) cells into lethally irradiated recipients (CD45.1). Donor contribution to peripheral blood and reconstitution to hematopoietic lineages was assessed at 4 and 8 weeks (Figure 4B). Strikingly, *U2af1*^mut/+^ donor cells displayed an engraftment failure compared to WT and *Tet2*^−/−^ bone marrow cells. This defect was also observed when *U2af1*^mut/+^ purified LSK were competitively transplanted (*n* = 3, WT = 13 and *U2af1*^mut/+^ = 14; Figure S2). Although we detected an increase in the frequency of LSK in *U2af1*^mut/+^ *Tet2*^−/−^ mice (Figure 4A), the repopulation defect was also observed when donor cells from *U2af1*^mut/+^ *Tet2*^−/−^ animals were transplanted (Figure 4C). Notably, multilineage reconstitution was achieved when WT and Tet2^−/−^ were transplanted and Tet2^−/−^ cells showed a higher reconstitution to the myeloid lineage in comparison to WT control (Figure 4D). These data suggest that mutant *U2af1* contributes to severe functional impairment of HSPC.

### 2.5. Exon Skipping Is the Alternatively Spliced Event More Frequently Observed

**Figure 5:**
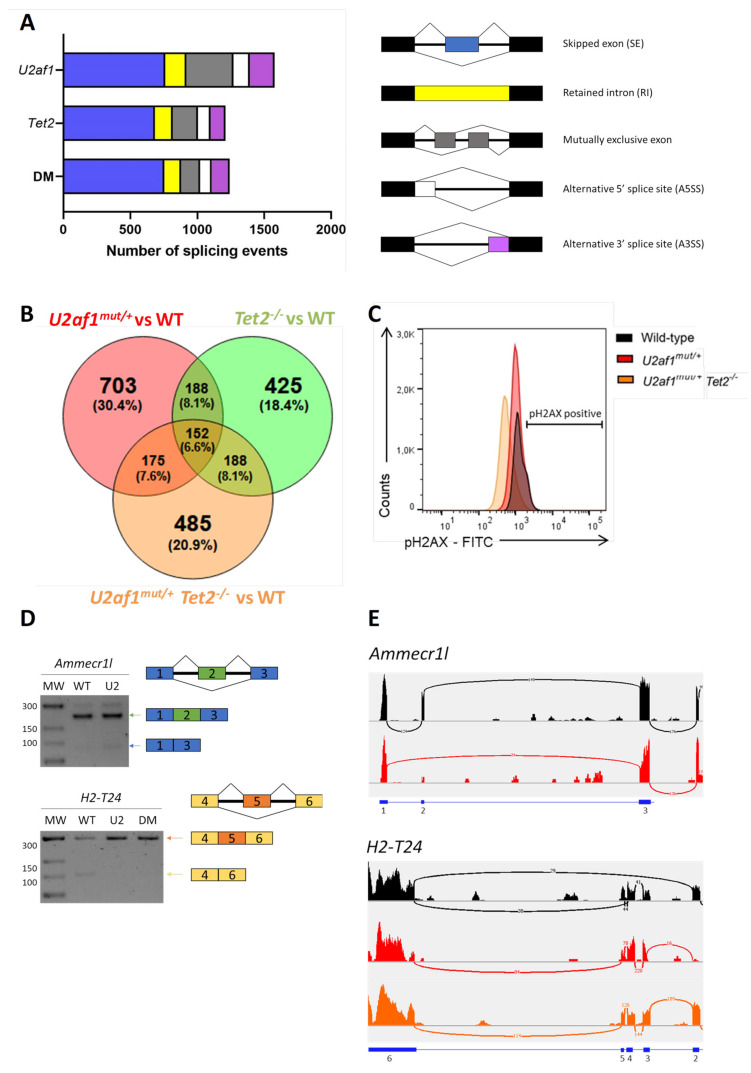
Aberrant splicing events by RNA sequencing. (**A**) Number of alternatively splicing events in U2af1^mut/+,^ Tet2^−/−^ and U2af1^mut/+^ Tet2^−/−^ mice. (**B**) Venn diagram showing the overlap of genes with alternative spliced events among comparison groups. (**C**) DNA damage accumulation in WT (*n* = 10), U2af1^mut/+^ (*n* = 4) and U2af1^mut/+^ Tet2^−/−^ (*n* = 6) represented as percentage of γ-H2AX+ cells in LSK, determined by flow cytometry. (**D**) Validation of aberrant splicing events identified by RNA-seq. RT-PCR bands using regular agarose gel. (**E**) Sashimi plots showing the Ammecr1l SE event in U2af1^mut/+^ samples and the H2-T24 SE in control samples. Black: WT; Red: U2af1^mut/+^; Orange: U2af1^mut/+^ Tet2^−/−^.

To explore the molecular basis of the impaired function of *U2af1*^mut/+^ and *U2af1*^mut/+^ *Tet2*^−/−^ cells, we performed transcriptome profiling of sorted LSK cells (WT (*n* = 5), *U2af1*^mut/+^ (*n* = 4), *Tet2*^−/−^ (*n* = 6) and *U2af1*^mut/+^ *Tet2*^−/−^ (*n* = 5)). Compared to WT controls, 181 genes were significantly dysregulated in *U2af1*^mut/+^ mice, 1505 in *Tet2*^−/−^ mice, and 555 in the *U2af1*^mut/+^ *Tet2*^−/−^ mice (*p* adjusted value < 0.05, Supplementary Table S4). Immunoglobulin-related genes were the most affected genes across all the genotypes. To determine the consequences in alternative splicing, we analyzed differential alternative splicing from sorted LSK using rMATS ^37^. Principal component analysis showed segregation of the genotypes, with *U2af1*^mut/+^ samples being more heterogeneous (Figure S3). Using pair-wise comparisons against WT control, we identified 1578 differentially spliced events in *U2af1*^mut/+^, 1211 in *Tet2*^−/−^ and 1242 in *U2af1*^mut/+^ *Tet2*^−/−^ (*p* value < 0.01, FDR < 0.01) (Figure 5A and Supplementary Table S4). This analysis showed that *U2af1* samples exhibit predominantly exon skipping events, which is in line with previously published results for mutated *U2AF1* [4,16], and that 152 alternative spliced genes were common among the different pair-wise contrasts (Figure 5B).

To seek downstream biological consequences, we performed enrichment pathway analysis. Kyoto Encyclopedia of Genes and Genomes (KEGG) analysis was conducted with the genes significantly mis-spliced in *U2af1*^mut/+^, *Tet2*^−/−^ and *U2af1*^mut/+^ *Tet2*^−/−^ mice compared to WT control mice, highlighting enrichment in cell processes, such as DNA damage, DNA repair and cell cycle, chromatin regulator and kinase pathway (Figures S4–S6). Of relevance, KEGG functional annotation in *U2af1*^mut/+^ *Tet2*^−/−^ group distributed mis-spliced genes in pathways similar to those found for *U2af1*^mut/+^. These ranked categories are in agreement with previous studies demonstrating that MDS-linked splicing signatures are related to cell cycle control, DNA damage and DNA repair [32]. To validate this further, we determined the phosphorylation status of histone γ-H2AX at Ser 139 in LSK cells as being a sensitive marker of DNA injury [33] and the first step in recruiting and localizing DNA repair proteins [34]. We found no differences in phosphorylated γ-H2AX in LSK cells from *U2af1*^mut/+^ and *U2af1*^mut/+^ *Tet2*^−/−^ compared to WT controls (Figure 5C). Next, we validated selected aberrant splicing events using RT-PCR in c-kit^+^ cells. We verified the *Ammecr1l* exon skipping event in U2af1^mut/+^ samples and the *H2-T24* exon skipping event in control samples (Figure 5D,E).

## 3. Discussion

MDS are complex and heterogeneous diseases in which a plethora of molecular pathways are disrupted. Massive sequencing studies of MDS patients have revealed mutations in genes with a role in RNA splicing and DNA methylation, among others. Some of these studies have linked certain splicing factor mutations to clinical phenotypes and biological features, for example, in the case of *SF3B1* [35], and have described patterns of concurrent mutations in patient samples [22]. However, given that pre-clinical modeling of MDS has proven difficult, there is a paucity of data related to how specific pathways influence one another to cause MDS. In the present study, we analyze the impact of mutant *U2af1* on the hematopoietic system in a novel CRISPR/Cas9 mouse model as well as its effect on MDS disease development in combination with *Tet2* deficiency.

Overall, our heterozygous mutant *U2af1* per se is not sufficient to cause MDS disease, even at an advanced age. This agrees with transgenic mice overexpressing U2AF1^S34^ [16] or U2AF1^Q157^ [17] and conditional knock-in *U2af1*^S34F^ mice [20] since these animals do not develop full-blown malignancy albeit presenting mild phenotypes with some MDS-like features. Of note, we observed that our mutant *U2af1* mouse model showed a defect in the reconstitution capacity in a competitive transplantation assay. Single mutant *U2af1*^mut/+^ and double mutant *U2af1*^mut/+^ *Tet2^−/−^* cells showed impaired engraftment with no donor chimerism detectable in peripheral blood of the recipient mice. The defect observed here is in line with the unanimous decreased competitive reconstitution capacity of HSC reported in all *U2af1-*mutant and *U2af1*-deficient mouse models [8,16,17,19,20]. This observation is puzzling in the field, given that splicing factor mutations are considered early events in MDS pathogenesis. It remains unexplained how mutant *U2AF1* clones can persist during disease evolution.

Human MDS bone marrow samples carry multiple mutations at diagnosis, suggesting that additional hits could be required to promote disease in our *U2af1* mutant model. Given that *TET2* is the most mutated gene in MDS and can co-occur with *U2AF1* mutations, we crossed mutant *U2af1* mice with *Tet2*-deficient mice. Several *Tet2* KO mouse models exist describing increased hematopoietic stem/progenitor cells, increased myeloid progenitors, and splenic extramedullary hematopoiesis at >1.5 year-old age [28,29,36,37]. Some of these findings, such as the increase in myeloid output, HSPC expansion, and splenomegaly, are also recapitulated in our model. One remarkable result from our double mutant mice is that there is no rescue of the impaired engraftment in mutant *U2af1*^mut/+^ *Tet2*^−/−^ cells, albeit the described enhanced ability of *Tet2*^−/−^ HSC, relative to WT HSC, to reconstitute hematopoiesis in vivo [37]. This is in agreement with the fact that deletion of *Tet2* in an *Srsf2* mutant background was also insufficient to rescue the impaired self-renewal capacity of *Srsf2* single mutant cells [38].

Transcriptomic analysis by RNA-seq showed that mis-spliced genes in mutant *U2af1^mut/+^* and *U2af1^mut/+^* Tet2^−/−^ mice belong to similar pathways, suggesting the lack of substantial differences between alternative splicing patterns in *U2af1*-mutated samples with and without *Tet2* alteration. Impairment of DNA repair has also been previously reported for splicing factor mutated MDS. *ERCC8* and *FANCM*, two genes involved in suppression/regulation of R-loop formation and in the DNA damage response, were found aberrantly spliced in *U2AF1* mutated MDS cases [39]. Remarkably, both genes were also affected in our *U2af1*^mut/+^ alternatively spliced signature. Regarding DNA injury, several studies associate R-loops-mediated DNA damage with *U2AF1* mutations [12,13]. However, we did not observe DNA damage in LSK cells as measured by γ-H2AX assay. Notably, 32 of the 140 genes alternatively spliced in a previously reported dataset from U2AF1^S34^ AML samples and U2AF1^S34^ and U2AF1^Q157^-expressing K562 cells [3] were in common with our *U2af1*^mut/+^ alternatively spliced signature. This would suggest that some selected mis-spliced target genes are shared across species, cell populations, and *U2AF1* mutations.

Together, this study improves our understanding of mutant *U2AF1* biology. Our findings indicate that heterozygous *U2af1*^mut/+^ mice display normal hematopoiesis albeit the impairment of hematopoietic cells to engraft in functional assays. Additionally, mutant *U2af1* combined with Tet2-deficiency does not suffice to initiate myelodysplastic syndrome. This suggests additional molecular alterations may be required to cause overt malignancy. A limitation of our approach is that complex combinatorial patterns of gene mutations cannot be achieved by intercrossing single mutant mice. Therefore, new pre-clinical modeling approaches would be needed to accurately recapitulate the molecular complexity of the MDS-spliceosome subgroup and to evaluate novel therapeutic targets.

## 4. Materials and Methods

### 4.1. Mice

Novel CRISPR/Cas9 U2af1 and Tet2 mice were generated as described in Supplementary methods. CD45.1 animals were purchased from Charles Rivers (Wilmington, MA, USA). The study was conducted according to the guidelines of the Declaration of Helsinki, and approved by the Ethics Committee of IIS La Fe (protocol code 2017/VSC/PEA/00200, date of approval 17 November 2017).

### 4.2. RT-qPCR

Total RNA from 10 × 10^6^ bone marrow cells was isolated using RNeasy Mini Kit (QIAGEN, Hilden, Germany). DNase treatment was applied using DNA-free DNase Treatment and Removal Reagents (Ambion, Austin, TX, USA). cDNA was synthesized from 1 µg of RNA using TaqMan Reverse Transcription Reagents (Thermo Fisher Scientific, Waltham, MA, USA) and oligo(dT) primers in a final volume of 25 μL. Real-time quantitative PCR reactions were performed in technical triplicates in 20 μL volume using AceQ SYBR qPCR Master Mix (Vazyme Biotech Co., Nanjing, China) and the respective primers (Supplementary Table S1) using a ViiA 7 Real-Time PCR System (Thermo Fisher Scientific). Primer set efficiency was first tested in a standard curve experiment. Relative expression was calculated from Ct obtained from Applied Biosystems ViiA 7 Real-Time PCR System software (Thermo Fisher Scientific). Since test and housekeeping primer set efficiencies differed >10%, we applied the corrective formula described in [40] to calculate the relative expression ratio. β-actin or *Hprt* expression was used for normalization (Supplementary Table S1).

### 4.3. Western Blotting

Cells were lysed in RIPA lysis buffer (Abcam, Cambridge, UK) supplemented with a complete Mini EDTA free protease inhibitor cocktail (Sigma-Aldrich Chemie GmbH, Munich, Germany). For TET2 Western blot, RIPA lysis buffer was supplemented with 0.5 µL/mL benzonase (Sigma) and 5 mM MgCl_2_. Equal amounts of total protein were separated on SDS-PAGE (Mini-PROTEAN electrophoresis system, Bio-Rad, Munich, Germany). Wet transference was performed on GE Healthcare Life Sciences Amersham Hybond PVDF membrane (Thermo Fisher Scientific). Blots were blocked at room temperature in 5% skimmed milk in TBST. Membranes were incubated overnight at 4ºC with primary antibodies and then incubated for 1h at room temperature with secondary antibodies. To detect U2AF1, we employed a rabbit monoclonal antibody (Cell Signaling Technology, Danvers, MA, USA, D6S3Q) at a 1:500 dilution. An anti-rabbit IgG H&L antibody (Abcam, ab6721) at a 1:5000 dilution was used as a secondary antibody. Anti-β-tubulin antibody (1:500 dilution; T4026, Sigma) was used as loading control. To detect TET2, we employed a polyclonal rabbit antibody against the N-terminus of Tet2 at a 1:1000. An anti-rabbit IgG H&L antibody (Abcam, ab6721) at a 1:5000 dilution was used as a secondary antibody. Anti-β-actin (Sigma, A3854) at 1:20000 or anti-lamin B1 (Cell Signaling Technology, D9V6H) at 1:1.000 was used as loading control. Protein signal was detected using ECL Western Blotting Substrate (Pierce) for U2AF1 or UltraScence Western Substrate (Bio-Helix, Keelung, Taiwan) for TET2.

### 4.4. Blood Count Analysis

Peripheral blood was collected from the facial vein of mice into an EDTA-coated tube (Sarstedt, Nümbrecht, Germany). Blood counts were determined using an automated blood cell counter (Sysmex XT-2000, Barcelona, Spain).

### 4.5. Flow Cytometry Analysis

HSC, progenitors, and mature cells from bone marrow or spleen were analyzed using a BD fluorescence-activated cell sorter (FACS) Canto II (BD Biosciences, Heidelberg, Germany). For myeloid progenitor analyses, we followed the immunophenotype scheme established by Pronk et al. [41]. Briefly, the bone marrow was isolated from femurs, tibiae and cristae of control and mutant mice. The hematopoietic cells were obtained by crushing bones with PBS + 2% FCS followed by filtering through a 70 µm nylon mesh (SPL Life Sciences). Red blood cells (RBCs) were lysed using Gey Solution (NH_4_Cl 8.3 g/L, NaHCO_3_ 1.0 g/L, EDTA 37 mg/L) and RBC-depleted cells were stained using monoclonal antibodies (Supplementary Table S2). To analyze hematopoietic cells in peripheral blood, RBC were lysed with BD FACS™ Lysing Solution (BD Biosciences) following the manufacturers’ instructions. Then, samples were stained using antibodies (Supplementary Table S2). Data analysis was performed with FlowJo v10 software (TreeStar Inc., Ashland, OR, USA).

### 4.6. Histological Analysis

Spleens were fixed in 4% paraformaldehyde and paraffin-embedded using a Leica ASP6025 tissue processor (Leica Biosystems, Wetzlar, Germany). Samples were sectioned using a Leica RM2245 microtome and stained with hematoxylin and eosin using a Dako CoverStainer (Agilent, Santa Clara, CA, USA). Stained sections were analyzed using a Leica DM 2000 microscope.

### 4.7. Cytospin Preparation

2 × 10^5^ bone marrow cells were spun on slides for 5 min at 600 rpm using Shandon Cytospin 3 Cytocentrifuge (Thermo Fisher Scientific). Slides were stained with May-Grünwald Giemsa solution (Sigma). 40× images were acquired using a Leica DMD 108 microscope. Scale bar = 50 µm.

### 4.8. Colony-Forming Unit Assays

For CFU-GM, 50.000 unfractionated BM cells were plated in MethoCult GF M3534 media in triplicate and colonies were scored on day 7. For BFU-E, 50.000 unfractionated BM cells were plated in triplicate in MethoCult SF M3436 media and colonies were counted on day 10. All media were from Stem Cell Technologies (Vancouver, BC, Canada).

### 4.9. DNA Damage

Flow cytometry was performed to analyze the phosphorylation status of histone H2AX (γ-H2AX Ser139) in LSK using H2A.X Phosphorylation Assay Kit (Millipore, Burlington, MA, USA). Data were analyzed using FlowJo v10 software (TreeStar Inc.).

### 4.10. LSK FACS-Sorting

Bone marrow cells from a pool of 3 mice were lysed with Gey Solution, c-kit enriched by LS columns (Miltenyi Biotec, Bergisch Gladbach, Germany), and stained with anti-mouse antibodies (Supplemental Table S2). Lin^-^Sca-1^+^c-Kit^+^ cells (LSK) were sorted using a FACSAria-III (BD Biosciences, Heidelberg, Germany).

### 4.11. Competitive Repopulation Assay

For competitive transplantation experiments, 1 × 10^6^ *U2af1*^+/+^ (thereafter WT), *U2af1*^mut/+^, *Tet2*^−/−^, or *U2af1*^mut/+^ *Tet2*^−/−^ unfractionated BM cells (CD45.2) were intravenously injected along with 1 × 10^6^ unfractionated BM competitor cells (CD45.1) into lethally irradiated (6 Gy × 2) 8–12 week-old mice (CD45.1). Donor chimerism in peripheral blood was assessed at 4 and 8 weeks after transplantation.

### 4.12. Behaviour Tests

The wire hang test and the beam balance test were performed as described in [42].

### 4.13. RNA Sequencing Library Construction

LSK (50,000 cells) from WT (*n* = 5), *U2af1*^mut/+^ (*n* = 4), *Tet2*^−/−^ (*n* = 6) and *U2af1*^mut/+^ *Tet2*^−/−^ (*n* = 5) mice were sorted into RLT buffer supplemented with β-mercaptoethanol. RNA extraction was performed using RNEasy Micro kit (QIAGEN). DNA was removed using on-column DNAse treatment. RNA integrity following isolation was assessed using Agilent High Sensitivity RNA ScreenTape Assay and Agilent 4200 TapeStation System. Samples with an RNA Integrity Number (RIN) value ≥7 were selected for further analysis. Low input directional RNA-seq libraries were prepared using NEBNext Ultra Directional RNA Library Prep Kit for Illumina (Illumina Inc., Cat N°. 7420, San Diego, CA, USA). Purified libraries were paired-end sequenced on the Illumina NovaSeq 6000 (Novogene, Beijing, China).

### 4.14. RNA Sequencing Analysis

The quality of raw RNA-seq data was assessed in fastq data files using FastQC. Reads were mapped to the reference mouse genome GRCm38 (mm10), with genome annotations from GENCODE vM24, using the splicing aware mapping software STAR (version 2.6.1c) [43]. In order to maximize junction read mapping, two passes of mapping were performed and uniquely mapped reads were selected for downstream analysis, following the STAR manual instructions. An average of 86% of reads per sample resulted uniquely mapped. Quality control specific for RNA sequencing experiments was also performed on BAM mapped files with RSeQC 2.6.4 software. Differential alternative splicing was assessed using rMATS 4.0.2 [44]. Splicing events associated with each pair-wise comparison were identified based on stringent parameters (false discovery rate (FDR) = 0.01 and difference in percent spliced-in (ΔPSI) = 0.1). rMATS output tables were further explored with the ‘maser’ package in R Bioconductor. Functional enrichments within lists of differentially spliced genes were identified with the DAVID tool (version 6.8) [45] and the enrichment cut-off p-value was set as <0.01. For differential gene expression analysis, counts of reads per gene were calculated with featureCounts function using Bioconductor ‘Rsubread’ package [46]. Differential gene expression was determined using DESeq2 (version 1.26.0) [47]. All the analysis and plotting functions were run under R version 3.6. RNA-seq data have been deposited in the ArrayExpress database at EMBL-EBI (www.ebi.ac.uk/arrayexpress) under accession number E-MTAB-10486.

### 4.15. Statistical Analysis

Data analysis was performed with GraphPad Prism version 8. Data are presented as mean ± SEM. Two-tailed Student’s *t*-test was used to determine statistical significance. We set statistical significance at *p* < 0.05.

## Data Availability

The data presented in this study are openly available in the ArrayExpress database at EMBL-EBI (www.ebi.ac.uk/arrayexpress) under accession number E-MTAB-10486.

## Supplementary Materials

The following are available online at https://www.mdpi.com/article/10.3390/ijms22136963/s1.
